# Combating Parasitic Weeds by Manipulation of Strigolactones Efflux Transporter

**DOI:** 10.1111/pce.15577

**Published:** 2025-04-29

**Authors:** Meicheng Zhao, Xianmin Diao

**Affiliations:** ^1^ Center for Agricultural Resources Research, Institute of Genetics and Developmental Biology Chinese Academy of Sciences Shijiazhuang China; ^2^ State Key Laboratory of Crop Gene Resources and Breeding, Institute of Crop Sciences Chinese Academy of Agricultural Sciences Beijing China

## Abstract

This is a commentary paper to Shi et al. (2025) and Ban et al. (2025). Resistance to *Striga* parasitism through reduction of strigolactone exudation. *Cell*. doi:10.1016/j.cell.2025.01.022; Manipulation of a strigolactone transporter in tomato confers resistance to the parasitic weed broomrape. *The Innovation*, 6(3). doi:10.1016/j.xinn.2025.100815.

Parasitic weeds from the *Orobanchaceae* family, including *Striga*, *Orobanche*, and *Phelipanche* species, can infect a wide range of staple crops, causing a severe yield loss and posing a significant threat to food security. *Striga* prefers to infect monocotyledonous cereal crops including maize, sorghum, millet and sugarcane. Outbreaks are most common in sub‐Saharan Africa, India and part of Asia, where the economic losses exceed $1 billion annually (Runo and Kuria 2018).

Host plants release strigolactone (SL) that act as signalling molecules to attract arbuscular mycorrhizal fungi (AMF) from the soil. This triggers a symbiotic relationship that facilitates the host's uptake of water and nutrients, especially under adverse conditions (Li et al. 2023; Han et al. 2025). Intriguingly, *Striga* also takes advantage of this mechanism, hijacking SL to trigger parasitism and invade the host (Trasoletti et al. 2022). *Striga* seeds are able to remain dormant in the soil for up to 10 years, but still can rapidly germinate once they detect the presence of SL secreted by host plants (Runo and Kuria 2018). Therefore, disrupting SL biosynthesis and/or exudation to the soil is a potential avenue for protecting crops from parasitic weeds. Recently, two independent studies have shown that knocking out SL efflux transporters can effectively reduce parasitic plants germination and their infection in host crops, without compromising crop yield (Ban et al. 2025; Shi et al. 2025).

## 1 Differential Selection of ABCG Transporter Members as SL Carriers in Monocots and Dicots

**Figure 1:**
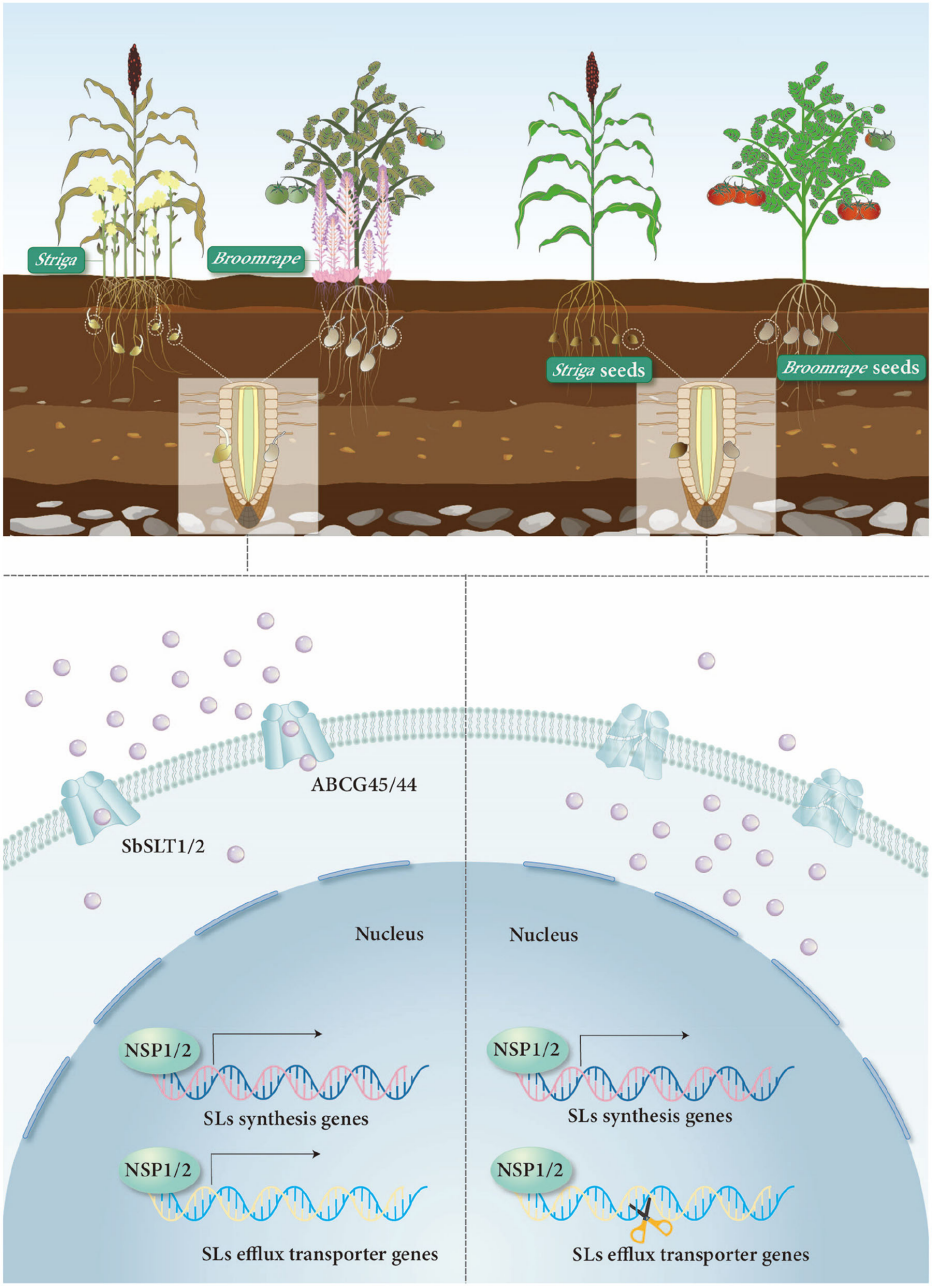
Enhancing plant resistance to parasitism through knockout of Strigolactone (SL) efflux transporters. Under phosphate (Pi)‐deficient conditions, the expression of SL biosynthesis genes and transport genes is upregulated by nodulation signalling pathway (NSP) transcription factors NSP1/2, resulting in increased SL secretion into the soil that triggers the germination of parasitic weeds. ABCG transporter members including SbSLT1 and SbSLT2 in sorghum, along with SlABCG45 and SlABCG44 in tomato, play a crucial role in this biological process. These transporters are localised at the plasma membrane and specifically expressed in root epidermal cells, where they export SL from the roots into the soil. Knocking them out restricts SL exudation and inhibits parasitic weed germination, protecting host crops from parasitism and yield losses.

Since direct inhibition of SL biosynthesis always brings multiple negative effects on crops, despite providing strong resistance to parasitic plants (Chen et al. 2018; Ban et al. 2025), Shi et al. (2025) adopted an alternative strategy by blocking SL secretion into the soil to inhibit *Striga* germination. Through integrated analysis of low phosphorus (Pi) conditions known to stimulate SL biosynthesis and rhizospheric secretion (Kun Yuan et al. 2023), and SL‐induced transcriptional profiles in sorghum, the authors identified two *ABCG* members, *ABCG36*/*SbSLT1* and *ABCG48*/*SbSLT2*, from the shared 121 differently expressed genes of the two treatments. They are paralogs of petunia *PhPDR1*, the first reported SL efflux transporter in plants (Kretzschmar et al. 2012). Shi et al. (2025) showed that SbSLT1/2 are plasma membrane‐located proteins, specifically expressed in root epidermal cells, and substantially induced by Pi deficiency or SL (GR24^5DS^), indicating their role as SL transporters in sorghum. Furthermore, they demonstrated that SbSLT1/2 have an ability to export SL to the extracellular matrix when expressed in yeast, oocyte, Arabidopsis and sorghum (Figure 1). Intriguingly, their closely related proteins, SbSLT1‐like and SbSLT2‐like, as well as sorghum PDR1 ortholog SbPDR1, were found to all lost this ability. In contrast, another study by Ban et al. (2025) showed that tomato PDR1 ortholog, SIABCG45 and SIABCG44, exhibited conserved SL efflux transporter activity similar to PhPDR1 in petunia. Collectively, these findings reveal a differential selection within ABCG family as SL transporter between monocots and dicots, likely due to major SL type specificity in different plants. For example, maize and tomato secrete stigol‐type and orobanchol‐type SL respectively. In addition, there is a difference in SL upward translocation between sorghum and tomato SL transporters. Knockout of *SIABCG45* and *SIABCG44* in tomato blocked more than half of the SL (GR24^4DO^) transport from root to shoot, resulting in more branching. However, knockout of *SbSLT1* and *SbSLT2* in sorghum seems not to influence SL (GR24^5DS^) upward transport. Indeed, SL content in the shoots of sorghum double mutants remained unchanged, although tiller number slightly increases, suggesting that other ABCGs or unidentified SL transporters may be involved in SL upward transport in sorghum.

Pi‐deficient soils significantly stimulate SL exudation, a critical mechanism promoting parasitic plant interactions. Ban et al. (2025) demonstrated that this process is primarily mediated by SL efflux transporters and their upstream regulatory components (Figure 1). Notably, they found that tomato *SIABCG45* exhibits remarkable upregulation (> 35‐fold) under Pi‐deficient conditions. Crucially, this induction requires the nodulation signalling pathway (NSP) transcription factors *NSP1* and *NSP2*, GRAS‐type regulators that concurrently govern Pi deficiency‐induced SL biosynthesis (Kun Yuan et al. 2023). This discovery establishes NSP1/2 as essential upstream regulators coordinating both SL biosynthesis and exudation under Pi limitation. Parallel observations in sorghum also revealed that Pi deficiency induced upregulation of *SbSLT1/2* transporters, though the regulatory mechanisms remain undefined. Whether NSP1/2 play a conserved function in sorghum needs to be further explored.

Shi et al. (2025) predicted the transport channel structure for SL in SbSLT1 and SbSLT2, identifying a phenylalanine residue that is essential for the export activity. Notably, this site is conserved among SbSLT1/2 orthologs in grass and other known SL transporters, such as PhPDR1 and SIABCG44, but not in SbSLT1/2‐like proteins. The researchers demonstrated that replacing the original isoleucine with this conserved phenylalanine is able to confer SL transporter activity to SbSLT1/2‐like proteins. The findings highlight the presence of the conserved phenylalanine residue as one of prerequisites for being an effective SL transporter. Based on the presence or absence of this residue in the SL transport channel, the authors were able to successfully identify maize SL transporters within ABCG family. This discovery provides valuable insights for identifying SL transporters in other plants with diverse SL types in the future.

## 2 The Great Potential for Breeding High‐Yield, High‐Resistance Crops by Manipulation of SL Efflux Transporters

Direct disruption of SL biosynthesis can confer strong resistance to parasitism, but it also causes abnormal plant architecture and yield losses (Ban et al. 2025). An alternative approach is to alter SL composition with a shift to parasitic weeds‐insensitive SL (Gobena et al. 2017; Li et al. 2023). Although this strategy appears not to have affected the normal development of plants and AMF colonisation, the impact on crop yield requires further evaluation in the field. Ban et al. (2025) identified *SIABCG45* as a natural selection target for combating parasitism through a genome‐wide association study (GWAS) of tomato varieties. They found that its knockout efficiently elevated the resistance to parasitism and fruit yield in the infested field. In line with this, knocking out *SbSLT1/2* in sorghum substantially inhibited *Strig*a germination and growth, consequently elevating yield under parasitism condition (Figure 1). Importantly, *SbSLT1/2* mutant plants showed normal development without obvious differences at grain yield from WT during the entire growth period. This is owing to that *SbSLT1* or *SbSLT2* deficiency, or both, did not cause substantial SL content changes in underground and aboveground parts of the plant, but solely led to a notable decrease in SL content in root exudates, further supporting their specificity in SL exudation. These two recent studies shed light on the effective management of parasitic weeds in agriculture by specifically regulating SL exudation.

## 3 Conclusions and Future Perspectives

In summary, the studies by Shi et al. (2025) and Ban et al. (2025) identified the different members within ABCG family as SL efflux transporter in monocots and dicots, and demonstrated that blocking SL exudation into soil by knocking out these SL transporters significantly elevates resistance to parasitic weeds and crop yield. However, since SL are common signalling molecule for parasitic weeds and AMF to establish colonisation with host plants, it is necessary to explore the impact of reduced SL secretion on AMF abundance and the consequent uptake of nitrogen and phosphorus by host plants, particularly in infertile soils. Notably, plants produce diverse SL (Li et al. 2023), suggesting a requirement for multiple transporters at least in ABCG family to accommodate structural variations. A natural genetic variation that shifts sorghum SL from *Striga*‐sensitive to ‐insensitive type could specifically inhibit *Striga* parasitism without affecting AMF symbiosis (Gobena et al. 2017). Therefore, it is of great interest to further investigate the change in SL profile, and preference for parasitism or symbiosis establishment when these SL efflux transporters are disrupted.

## Conflicts of Interest

The authors declare no conflicts of interest.

## Data Availability

The data that support the findings of this study are available from the corresponding author upon reasonable request.
